# Antioxidant Molecules in the Human Vitreous Body During Prenatal Eye Development

**DOI:** 10.3390/antiox14091041

**Published:** 2025-08-23

**Authors:** Ina G. Panova, Tural Galbinur, Alexander S. Tatikolov

**Affiliations:** 1 International Scientific and Practical Center of Tissue Proliferation, 14/19 Prechistenka St., Moscow 119034, Russia; 2 Department of Ophthalmology, Azerbaijan Medical University, 163 Samad Vurgun Street, Baku AZ1022, Azerbaijan; tqelbinur@amu.edu.az; 3 N.M. Emanuel Institute of Biochemical Physics, Russian Academy of Sciences, 4 Kosygin St., Moscow 119334, Russia

**Keywords:** vitreous body, reactive oxygen species, antioxidants, human eye, prenatal development

## Abstract

The structures of the developing eye may be damaged as a result of the impact of reactive oxygen species (ROS) interacting with different cellular components. The antioxidant molecules found in the eye, especially in the vitreous body—the largest component of the eye, playing a crucial role in the formation of structures and functions of the developing eye—provide protection to the eye tissues from ROS. This review considers various antioxidant molecules (ascorbic acid, lutein, bilirubin, uric acid, catecholamines, erythropoietin, albumin, and alpha-fetoprotein) that have been found in the human vitreous body during the early stages of pregnancy (10–31 weeks of gestation) and their functions in the development of the eye. The presence of some molecules is transient (lutein, AFP), whereas a temporal decrease (albumin, bilirubin) or increase (ascorbic acid, erythropoietin) in the concentrations of other antioxidants is observed. Since the actual overall content of antioxidants in the developing vitreous body is probably much higher than that found to date, further research is needed to study antioxidants there. It is especially important to study the antioxidant status of the vitreous body at the earliest stages of its development. Antioxidants found suggest their use for the prophylactic of ocular diseases during pregnancy and finding new antioxidants could create an additional opportunity in this regard.

## 1. Introduction

The eyes are the most important human sense organs, through which humans receive most of the information from the outside. The normal functioning of the eyes is largely determined by their correct embryonic development, without significant deviations and disorders. One of the adverse factors in the body is reactive oxygen species (ROS)—reactive radicals and molecules, which, if in excess, can cause developmental violations of the eye, leading to damage to the visual system and even blindness. Protection of the eye structures from ROS is provided by the antioxidant system—a number of molecules present in the eye, which, interacting with ROS, deactivate them. The human eye has a complex composition and consists of many structural components, the most voluminous of which is the vitreous body (or the vitreous)—a vital component of the ocular media. It is a transparent, colorless, gel-like substance that makes up about 4/5 of the eye’s volume. It occupies the cavity between the retina and lens and is extremely hydrated (contains about 99% water). Normally, after birth (in adult humans), the vitreous is free of blood vessels. The sole cells of the vitreous are hyalocytes. Proteins, amino acids, collagens, hyaluronic acid, electrolytes, and other substances are the main molecular constituents of the vitreous [1,2,3].

The structure and functionality of the eye are significantly influenced by the vitreous. It maintains the form and tonus of the eyeball, softens mechanical impacts, thus protecting the retina and lens from damage, regulates intraocular pressure, and participates in the metabolism of eye tissues. It also maintains normal homeostasis of the retina and lens, and is of importance in maintaining vital activity of these tissues, having anti-inflammatory, antiangiogenic, and antioxidant properties [2,3,4,5,6].

At the early stages of prenatal development, the vitreous plays an important role as a factor of intraocular pressure, which is necessary for the coordinated growth of all parts of the eye and ocular tissue differentiation [7,8,9,10].

The formation of the vitreous is a complex process and occurs in close interconnection with the retina and the lens. In the course of prenatal human development, the vitreous dynamically changes both morphologically and biochemically and includes a number of successive stages.

Ida Mann, in her classical monograph [11], divides the development of the vitreous into three periods. The first period lasts up to 6 weeks of pregnancy (13 mm embryo) and ends with the closure of the fetal (choroidal) fissure. During this period, the primary vitreous is formed. The second period lasts from 6 weeks up to 12 weeks of pregnancy (from 13 mm up to 60 mm embryo) and includes the development of the secondary vitreous. The third period—after the 12th and up to the 28th week of pregnancy—is characterized by the formation of the tertiary vitreous [11]. When the optic cup is formed, its cavity becomes the vitreous cavity. It is filled with collagen fibers and fibrils, derivatives of the retina and lens cells, marking the beginning of the formation of the primary vitreous. Mesenchymal cells surrounding the optic cup penetrate into the forming vitreous cavity through the fetal fissure, together with the hyaloid artery (arteria hyaloidea), growing here and through the edge of the optic cup. The retina, lens, hyaloid vessels, and mesenchyme participate in the formation of the extracellular matrix of the primary vitreous [11,12].

A characteristic feature of the primary vitreous is the presence of a network of hyaloid vessels; therefore, it is called the vascular or cellular vitreous [1,11]. The first stage of vitreous formation ends with the formation of the hyaline capsule of the lens and the closure of the fetal fissure. In humans, this occurs on the 6th week of embryonic development [11,13].

The secondary vitreous, which is entirely derived from the retinal cells, consists of very thin, tightly packed fibrils. It is formed between the 6th and 12th weeks of gestation and marks the second stage of vitreous development. At the 10th week, all vessels of the hyaloid system are morphologically present in human fetuses, and from this moment, the first signs of their regression appear [14]. The hyaloid vessels, which are necessary for nutrition of the developing lens and retina, after serving their role, gradually disappear [15,16,17,18]. The process of regression of hyaloid vessels is completed by the 36th week of pregnancy or by the birth of the child [11,19,20].

The area of more compact collagen fibrils, being at the border between the primary and secondary vitreous fibrils—the condensation line—forms the walls of Cloquet’s canal [11]. The third stage of vitreous formation begins in human fetuses from the 13th week and is characterized by the development of the tertiary vitreous (zonule, or zonule of Zinn). The zonule develops in parallel with the differentiation of the lens and the development of the ciliary body [13].

An essential biological task is the study of the development of the vitreous, specifically the characteristics that define its functions. Using histochemistry and immunohistochemistry, the developmental dynamics of collagen and glycosaminoglycans, and temporal changes in collagen composition, were demonstrated in the vitreous of embryos and fetuses of humans [21,22,23].

Hyaluronic acid (nonsulfated glycosaminoglycan), the main component of the vitreous, was found in it from the 5th week of embryonic development. Sulfated glycosaminoglycans, on the other hand, are temporarily present in the vitreous from the 6th to 40th week of gestation. Collagens also change during human embryonic development: type III collagen was found in the vitreous up to the 8th week, whereas type II collagen becomes the main collagen from 8 weeks throughout the further human life [21]. The vitreous of 19-week human fetuses contains type IV and type XVIII (heparan sulfate proteoglycan) collagens [24]. The fetal vitreous was also shown to contain opticin, a member of the extracellular matrix family that belongs to leucine-rich repeat proteins/proteoglycans [25]. The regulation of fibril diameter and interfibrillar spacing is the biological role of opticin in vivo [26]. The vitreous is composed of a network of thin collagen fibrils (collagens of types II, IX, and V/XI), which are important for maintaining its structure. Opticin plays a role in the fibrillogenesis of collagens for creating the vitreous gel and may also play a role in preserving the distance between the vitreous collagen fibrils [2].

During embryonic and fetal development, the human eye requires special conditions for its development into a full-fledged visual organ. At present, modern molecular biological approaches to the study of the developing eye are in progress, allowing the identification of various phenotypes of the eye during its development [27].

Overexpression of ROS (oxidative stress) is harmful to cells and tissues, and leads to interruption of cell proliferation, damage to lipids, proteins, nucleic acids, etc., which are very important for normal eye development. Therefore, the antioxidant system protecting from ROS is one of the mechanisms for its correction. The retina and lens require special antioxidant protection due to high polyunsaturated fatty acid content in cell membranes. The proximity to these tissues of blood vessels (hyaloid vessels of the vitreous during growth and regression, vessels of tunica vasculosa lentis around the developing lens, choriocapillaries of the choroid, developing vessels of the retina itself) carrying oxygen creates a risk of tissue damage caused by oxidative stress. The antioxidant system of the vitreous, which is in close contact with the retina and lens, can provide protection of developing eye tissues from oxidative stress. The content of antioxidants in the mature vitreous and their role in the healthy eye and in various eye diseases were considered in the review of Ankamah et al. [5]. Note that the health of the adult eye is largely determined by its proper development, which essentially depends on the antioxidant status of the tissues of the developing eye, in particular, the vitreous. However, in human prenatal development, the antioxidant status of the vitreous has not been considered to date. Although some molecules with antioxidant properties have been found in the vitreous of human fetuses, their antioxidant capacity and its possible impact on the whole eye, which is the topic of this review, have not been addressed.

This review considers antioxidant molecules found in the developing human vitreous during early stages of pregnancy (prenatal development, 10–31 weeks of gestation) and their possible functions in the developing eye.

The literature search was carried out, collecting information on antioxidant molecules found in the vitreous of the human eye (ascorbic acid, bilirubin, carotenoids, uric acid, catecholamines, erythropoietin, albumin, alpha-fetoprotein, etc. and those mentioned in [5]) from various Internet databases (Web of Sciences, Scopus, PubMed, Medline, Ovid). The search was also carried out using the following keywords: vitreous body; reactive oxygen species; antioxidants; human eye; and prenatal development.

The structures of low molecular weight molecules were created using ChemDraw Ultra 9.0, and the structures of proteins were taken from the Protein Data Bank (PDB).

## 2. Redox System in the Body

Normally, the body maintains a balance between oxidative and reductive processes (redox homeostasis). The redox balance provides the consistency and functioning of cells, tissues, and organs. All the main processes in the body are regulated by the redox system [28,29,30].

The oxidant system, represented by low-molecular-weight ROS, is responsible for oxidative reactions. Superoxide radical-anion (O_2_^−•^), hydroperoxyl radical (HO_2_^•^), hydroxyl radical (OH^•^), hydrogen peroxide (H_2_O_2_), hypochlorous acid (HOCl), singlet oxygen (^1^O_2_), nitric oxide (NO), and peroxynitrite (ONOO^−•^) are highly reactive oxygen-containing free radicals and molecules that are included in ROS. They are produced by specialized enzymes (e.g., nitric oxide synthase, NADP oxidase) or appear as byproducts of the mitochondrial respiratory chain. Reactive nitrogen species (RNS) are produced from nitric oxide via its interaction with O_2_^−•^ giving ONOO^−•^ [30,31,32,33,34].

ROS have strong destroying ability, which is manifested in oxidative destruction of lipids, proteins, nucleic acids, and other biomolecules [29].

Alternatively, despite their instability, ROS alter the expression of transcription factors and the activity of several enzymes, including proteinase, phosphatase, and phospholipase, which results in global stable alterations in gene activity and cell metabolism. Despite their prominent role in numerous physiological processes and signaling pathways, the molecular mechanisms of ROS regulation activity remain insufficiently studied [35].

Antioxidants are substances that slow down or prevent oxidative damage of ROS to cells and their components. Low-molecular-weight-reducing molecules (such as vitamin C, glutathione, cysteine, taurine, uric acid, carotenoids, etc.) and enzymes neutralizing ROS (such as catalase, superoxide dismutase, glutathione peroxidase, and peroxyredoxins) or reduce oxidized forms of proteins and lipids (such as the glutathione enzyme system) are components of the antioxidant system [36]. Antioxidants can be considered endogenous or exogenous. Endogenous antioxidants are formed in the human body, while exogenous antioxidants originate from human food. Both types of antioxidants can be found in food or can be taken as dietary supplements. To establish or preserve redox equilibrium, endogenous and exogenous antioxidants can operate synergistically. Note, however, that under certain conditions, antioxidants can work as pro-oxidants, that is, substances that stimulate the generation of ROS. This relates, for example, to ascorbic acid (at low concentrations in the presence of small amounts of metal ions with variable valence [37,38]) and uric acid (essentially within cells) [39].

ROS and antioxidants are the main parts of a redox system, which maintains the equilibrium concentrations of ROS in the body [40]. One of the most important roles of redox systems is to trigger various signaling pathways that control fundamental processes in the body: proliferation, differentiation, apoptosis, cell protection, anti-inflammation, and immune response (redox signaling) [41,42].

An imbalance between the generation and decomposition of ROS toward their accumulation leads to oxidative stress, which may cause cell and tissue damage [29,30,34,43,44]. Additionally, excessively accumulated oxidized proteins can aggregate and result in protein unfolding [45]. Growing ROS can also decrease the nitrogen oxide bioavailability [46].

For normal development of the body, including the eye, a redox balance is necessary in its tissues, in particular, in the vitreous, which is ensured by the presence of antioxidants in it during prenatal development.

## 3. Antioxidant Molecules in the Vitreous of the Human Fetal Eye

### 3.1. Ascorbic Acid (Vitamin C)

**Figure 1:**
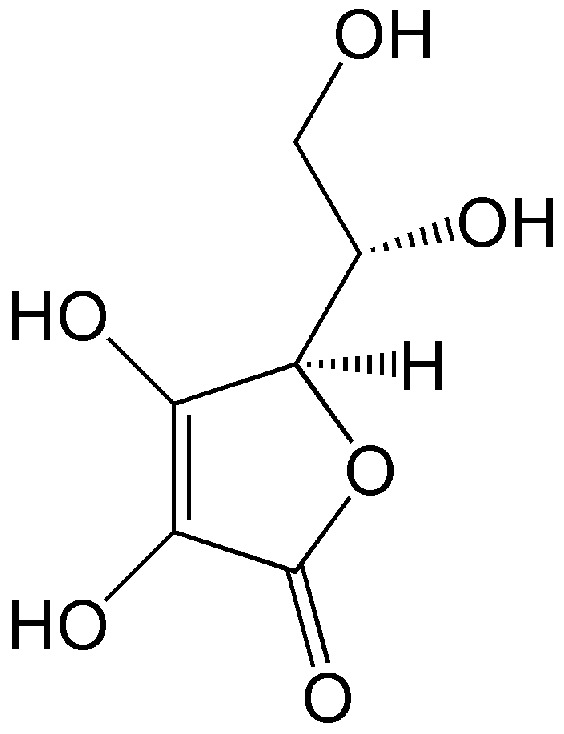
Structure of vitamin C (L-ascorbic acid), an active antioxidant. Due to the presence of four OH groups, it is soluble in water.

An important antioxidant compound present in the developing vitreous is vitamin C (L-ascorbic acid, Figure 1). It is an organic compound soluble in water, occurring in living organisms and in diet products. Humans do not synthesize ascorbic acid but receive it exclusively from food [47,48,49,50,51]. Ascorbic acid enters the vitreous from the blood plasma due to active transport from the ciliary epithelial bilayer [52,53].

Ascorbic acid at physiological pH is mainly present as the ascorbate monoanion [54]. At physiological concentrations in the blood plasma of a healthy human (about 40–80 μmol/L), ascorbic acid functions as an active endogenous antioxidant: it effectively reacts, in particular, with radicals O_2_^•−^, HO^•^, RO^•^, and ROO^•^, as well as with H_2_O_2_, ^1^O_2_, and NO, turning them into inactive compounds [55,56]. Additionally, ascorbic acid protects the reduction ability of other antioxidant molecules such as α-tocopherol by reducing its oxidized radical form (α-TO^•^). This allows regeneration of the antioxidant, prolongs its lifetime in the lipid phase, and helps to remove radicals from the lipid phase into the aqueous phase [37,57].

In the vitreous of human fetuses, ascorbic acid was measured from 10 to 24 weeks of gestation, and it has been shown that with fetal age, the concentration of ascorbic acid gradually increases from 0.32 mg per 100 g of vitreous (18 µmol/L) at 10 weeks to approximately 2 mg per 100 g of vitreous (114 µmol/L) in 24-week fetuses, which is comparable to its concentrations in the vitreous of the adult human eye (2.7–2.9 mg per 100 g, that is, ~160 µmol/L) [58].

The importance of ascorbic acid for the developing eye can be assumed from its properties. In particular, ascorbic acid is known to perform important biological functions in the body as a reducing agent, a coenzyme of some metabolic processes, and an effective antioxidant (electron donor) [59]. It is able to modulate gene expression and is involved in the processes of cellular differentiation [59].

As an antioxidant, ascorbic acid prevents lipid peroxidation–oxidative damage to lipids. Ascorbate in the vitreous consumes oxygen released at the vitreous–retinal interface and protects against intraocular oxidative stress and the development of nuclear cataracts [60].

Ascorbic acid, being a cofactor of the enzymes prolyl hydroxylase and lysyl hydroxylase, is necessary for the synthesis of collagen. It is involved in post-translational hydroxylation of collagen and is also necessary for the formation of collagen fibers [37,61]. Ascorbic acid was shown to greatly increase the transcription of genes of type I and type III collagens in fibroblasts of human skin, but it only slightly stimulated the transcription of type IV collagen [62].

Ascorbic acid in the vitreous has a stabilizing effect on collagen fibrils. Under the conditions of culturing hyalocytes—the only cells in the vitreous—it was shown that adding ascorbic acid to the culture medium enhances the proliferation of hyalocytes and increases collagen synthesis by hyalocytes. Therefore, ascorbic acid acts as a regulator of hyalocyte production of extracellular matrix and hyalocyte proliferation [63].

The stimulation of catecholamine production in neurosecretory cells by ascorbic acid is well recognized [64,65]. Because of its antioxidant ability and capacity to promote collagen synthesis, ascorbic acid inhibits angiogenesis and lowers blood vessel permeability [66,67,68].

Thus, the presence of necessary concentrations of ascorbic acid in the prenatal vitreous is essential for such important processes as collagen biosynthesis by hyalocytes and formation of collagen fibrils. The antioxidant and antiangiogenic properties of ascorbic acid are important for the protection of the retina, lens, and vitreous from damage and for maintaining the transparency of the vitreous gel and lens during eye development.

### 3.2. Lutein

**Figure 2:**
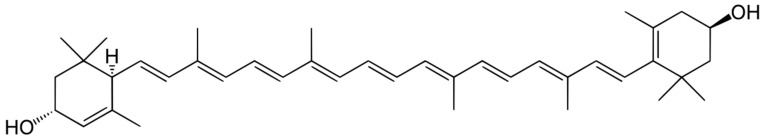
Structure of lutein. Due to the presence of a long polyenic chain, lutein effectively quenches singlet oxygen and is an active antioxidant.

Another important antioxidant found in the vitreous of human fetuses is lutein (Figure 2).

Lutein belongs to the xanthophyll group of carotenoids—pigments produced by plants and photosynthetic bacteria [69,70,71]. It is a potent antioxidant due to its peculiar structure—the extended system of conjugated double bonds (Figure 2). The lutein molecule contains two terminal hydroxyl groups, which makes it more hydrophilic. Like other xanthophylls, zeaxanthin and lutein efficiently quench singlet oxygen and lipid peroxyl radicals [72,73,74].

Unlike the vitreous of adult humans, where carotenoids were not found [75], lutein has been detected in the vitreous of the human eye during pregnancy, as a result of a detailed study from 12 to 28 weeks of gestation using HPLC and mass spectrometry [76]. The lutein content in the vitreous reaches a maximum (900–1000 ng/g, that is 1.6–1.8 µmol/L) within 17–22 weeks of pregnancy and gradually decreases by the 24–28th weeks (~4 ng/g, that is 0.007 µmol/L, at 24–28 weeks). In human fetuses at 30–31 weeks of age, carotenoids are no longer detected in the vitreous, as in the vitreous of an adult [76].

At approximately the same time, lutein was detected in the retina of 17–22-week-old human fetuses, and especially in the developing macula [77]. Such an early appearance of lutein in the vitreous and the retina suggests that there is a causal relationship between these events. The detection of lutein in the fetal vitreous gives arguments for supposing a substantial morphogenetic role of lutein in the formation and differentiation of the macular area of the retina. It is possible that the vitreous in prenatal eye development is an additional source of lutein for the developing macula, which also protects the entire retina from oxidative stress.

It has been shown that lutein has neuroprotective and anti-apoptotic effects [78]. Lutein can directly protect retinal ganglion cells from oxidative stress caused by H_2_O_2_ and hypoxia induced by cobalt chloride in vitro [79]. Decreasing the ROS production in the retina of diabetic mice, lutein blocks the signaling pathway related to NFκB and reduces the oxidative marker levels, which leads to retaining functionality of the retina [80].

Carotenoids are not produced in humans; instead, they are obtained through diet, and the placenta transfers these compounds to fetuses [81,82]. Carotenoids bound to serum albumin are transferred to target tissues as a complex “albumin-carotenoids” [82,83]. Alpha-fetoprotein is also a carrier protein for carotenoids [84].

Although the mechanisms of lutein uptake and stabilization within the fetal vitreous are still unknown, it cannot be ruled out that, functionally, lutein found in the vitreous also protects the developing vitreous itself from ROS. This assumption is related to the fact that ROS are formed during the regression of hyaloid vessels, and therefore, there is a risk of vitreous damage. This is also supported by the fact that, in addition to lutein, oxidized forms of lutein are found in the vitreous during this period of prenatal development [76].

Lutein and zeaxanthin are assigned an important protective function in the adult eye. They act as a light-cutting filter that protects visual cells and the lens from the damaging effects of blue light, and as effective antioxidants that protect cells from lipid peroxidation and from the damaging effects of ROS [85,86,87,88]. Since fetal development in the mother’s body occurs in the absence of light, this excludes light as a damaging factor in the development of the retina and lens. Nevertheless, the detection of lutein and its oxidized products in the human eye during pregnancy [76,77] suggests that it probably plays a significant role in the development of eye structures and functions.

Some physical and chemical properties, as well as biological functions of carotenoids, are considered in the reviews [89,90]. The presence of oxidized forms of carotenoids (lutein and zeaxanthin) in the retina indicates that carotenoids, supplied with food, can act as antioxidants [91,92].

It is known that, unlike adults, infants lack autoregulation of blood flow in the retinal vessels and choroids. These vessels release excess oxygen into the retina, which leads to its hyperoxygenation, generation of free radical processes, and lipid peroxidation of cell membranes [93]. Antioxidant protection of the retina is especially important during this period. Carotenoids are able to prevent peroxidative destruction of polyunsaturated fatty acids [89], and, apparently, this property of carotenoids is essential for preserving the membranes of developing neurons and their sprouts during retinal development. Another function of lutein is that lutein molecules, due to the presence of a polyene chain, protect the retina from the damaging effects of singlet oxygen (quench singlet oxygen) [94]. From these facts, it is possible to conclude that in prenatal human development, lutein can play a significant role in preserving the cells of the retina and lens from the destructive impact of singlet oxygen during the period when the active formation of definitive retinal vessels and regression of hyaloid vessels occurs.

A characteristic property of carotenoids is their ability to enhance intracellular connections between glial and neuronal cells of the retina by increasing the number of intermediate contacts. The channels of intermediate contacts serve to conduct electrolytes, nutrients, and secondary messengers transferred from the cytoplasm of some cells to others by diffusion, thus maintaining tissue homeostasis [95,96,97].

The property of carotenoids to participate in the regulation of apoptosis [98] suggests that carotenoids may serve as one of the factors in the regulation of apoptosis of hyaloid vessel cells during their regression and of retinal ganglion cells during prenatal development.

The data presented may be crucial for comprehending the role of carotenoids as antioxidants in the protection of the eye during development, and for revealing the pathogenesis of eye diseases such as congenital cataracts, retinopathy of prematurity, and defects in macular development.

### 3.3. Bilirubin

**Figure 3:**
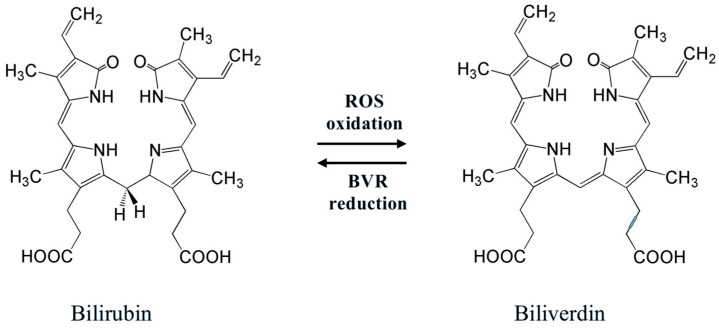
Redox equilibrium bilirubin–biliverdin, which determines bilirubin antioxidant activity. Two meso-H atoms of a bilirubin molecule easily react with ROS, converting them into inactive forms.

An important antioxidant found in the vitreous of fetuses is bilirubin (Figure 3 left)—a yellow pigment of bile. It is an open-chain tetrapyrrole, a product of catabolism of heme-containing proteins (mostly hemoglobin). Oxidative destruction (involving heme oxygenase-1, HO-1) of a porphyrin unit in heme affords biliverdin, which can be reduced back (involving biliverdin reductase, BVR) to bilirubin (Figure 3) [99,100].

Bilirubin is a potent serum antioxidant playing an important role in protection from ROS. Its antioxidant action consists of redox reactions with ROS to give biliverdin, which is again reduced to bilirubin by BVR (Figure 3). Incorporating into cells, bilirubin can protect cell membranes from oxidation of lipids by ROS [101,102,103]. The physiological redox homeostasis of the vascular endothelium is determined by the antioxidant activity of bilirubin, which may be a dynamic factor in endothelial function [104]. In addition, erythroid-2-related factor 2 (Nrf2), which takes part in antioxidant activities in cells of mammals, can be activated by excessive levels of bilirubin, causing oxidative stress [105]. By triggering several antioxidant genes, such as HO-1, NADPH quinone oxidoreductase, glutamate-cysteine ligase catalytic and modulatory subunits, glutathione S-transferase A2, glucose-6-phosphate dehydrogenase, 6-phosphogluconate dehydrogenase, and the cystine transporter SLC7A11, this transcription factor maintains redox homeostasis [106].

Bilirubin has also been demonstrated to possess additional biological characteristics, such as anti-inflammatory, immunomodulatory, cytoprotective, and neuroprotective properties, in addition to its antioxidant capacity [107,108,109,110,111]. It has been shown to behave as a signaling molecule that can activate the transcription factor known as peroxisome proliferator-activated receptor alpha (PPARα) [112]. Bilirubin is also thought to act as a hormone, and this mechanism may be responsible for some of its biological actions [113,114,115,116].

The measurement of bilirubin in the vitreous of fetuses from 17 to 31 weeks of gestation showed its presence at all stages of development studied. At earlier stages, from 17 to 19 weeks, the bilirubin concentrations (on average, 8.67 µmol/L) are significantly higher than at subsequent stages of development from 20 to 31 weeks (on average, 1.37 µmol/L). Hence, the level of bilirubin in the vitreous from 17 to 19 weeks exceeds the level from 20 to 31 weeks by about 6.3 times [117].

In the work of Weiner [118], the measurements of serum bilirubin concentration in 20–38-week human fetuses showed that it did not change significantly with gestation age (mean 1.4 ± 0.3 mg/dL [118], which corresponds to 23.9 µmol/L). Hence, from 20 to 31 weeks, the concentration of bilirubin in the vitreous is about 17.4 times lower than that in serum.

Relatively higher concentrations of bilirubin found in the vitreous at 17–19 weeks of gestation [117] are likely required for continuing retinal development: neuronal differentiation, axon growth, formation of synaptic connections, and retinal vessel sprouting. During this time, new fibers differentiate in the lens. Hyaloid vascular regression and the formation of the secondary vitreous are ongoing [11]. A comparatively high level of bilirubin in the vitreous can help to provide the antioxidant protection needed for all these processes. As an endogenous antioxidant, bilirubin most likely plays a role in the protection of the developing retina and lens from severe oxidative destruction in neurons of the retina and fibers of the lens. The eye growth and development processes are nearly finished by the 20th week of pregnancy; the layers of the retina are nearly completely formed, and, based on its morphology, the retina resembles the adult pattern [11]. Meanwhile, the bilirubin concentration in the vitreous drops sharply [117]. On this basis, it can be concluded that the surrounding tissues regulate and control the bilirubin content in the vitreous. Bilirubin most likely plays a role in the antioxidant protection of the developing tissues of the eye.

### 3.4. Uric Acid

**Figure 4:**
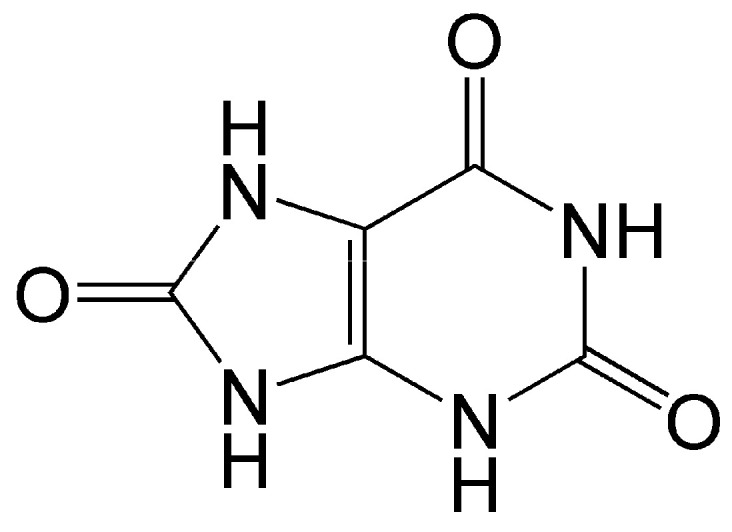
Structure of uric acid. As an antioxidant, it enters into one-electron redox reaction with active oxy radicals and effectively quenches singlet oxygen.

Uric acid is the end metabolic product of purine nucleotides (guanine and adenine) in humans and some higher primates (Figure 4).

Uric acid is diprotic; at physiological pH, monourate predominates in solution. As an antioxidant, it enters into one-electron redox reaction with peroxyl and other oxy radicals [119]. It is a strong quencher of singlet oxygen and regulator of oxidative stress, and is able to protect organs and tissues from damage caused by free oxygen radicals [120,121]. It is a component of blood serum, is present in body fluids and tissues, and, in addition to its antioxidant properties, serves as a signaling molecule for a number of pathological conditions such as gout, hyperuricemia, arthritis, and cardiovascular and renal disease [120,121,122]. Uric acid is an endogenous danger signaling molecule in case of cell damage, which stimulates the immune system by activation of T cells [123,124].

It has been found, in particular, that the antioxidative action of uric acid in blood plasma can be activated by ascorbic acid by repairing urate radicals formed upon oxidative stress [125]. Uric acid inhibits oxidative degradation of hyaluronic acid by ascorbate [126]. This ability of uric acid to protect hyaluronic acid from degradation [126] is very important for the vitreous, in which hyaluronic acid is one of the main components [2].

Uric acid exhibits neuroprotective properties, which have been shown after experimental cerebral ischemia [127].

Uric acid was detected in the human vitreous during prenatal development. Its concentration varies greatly (from 37 to 248 μmol/L), and age dependence is absent (analysis was carried out at stages from 17 to 31 weeks of gestation) [128]. This may be due to both purine metabolism and maternal nutrition during pregnancy.

Uric acid was also found in the adult vitreous, with strong variation of its concentration (from 77 to 452 μmol/L) [129].

Physiological concentrations of serum uric acid (140–430 µmol/L) display anti-inflammatory and chondroprotective effects both in vitro and in vivo [130]. However, high uric acid concentrations in serum (>70 mg/L in men and >60 mg/L in women) or in urea may result in gout and uric acid nephrolithiasis, hypertension, chronic renal disease, and cardiovascular disease, including several diseases linked to increased oxidative stress [39,131,132,133]. The dual function of uric acid as an anti- and/or pro-oxidant was discussed [131].

Thus, uric acid can have an important physiological role in the eye during pregnancy. The antioxidant properties of uric acid protect developing eye tissues from oxidative stress, which ensures their normal development.

### 3.5. Catecholamines

**Figure 5:**
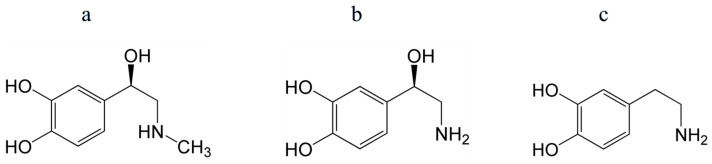
Structures of epinephrine (adrenaline) (**a**), norepinephrine (noradrenaline) (**b**), and dopamine (**c**). Their antioxidant properties are mostly determined by a catechol (phenol) group.

Important molecules present in the body include catecholamines, such as adrenaline, noradrenaline, and dopamine, which are derived from the amino acid tyrosine (Figure 5). These are a class of molecules that act as neurotransmitters and hormones in various organs.

Catecholamines are effective antioxidants—radical and singlet oxygen scavengers [134,135]. In particular, dopamine has been shown to react effectively with radicals OH^•^, HO_2_^•^, and presumably ROO^•^ [136]. The superoxide scavenging activity of dopamine and norepinephrine was found to be higher than that of ascorbic acid [135].

Catecholamines are determined in human tissues even at early stages of fetal development [137]. They are essential in controlling physiological functions and the onset of certain diseases (neurological, mental, endocrine, and cardiovascular) [138]. The physiological action of catecholamines is due to their capacity to attach to specific receptors located on the membrane of effector cells. The effects of catecholamines on mammalian retinas have been well examined. Dopamine and adrenaline were found in amacrine neurons that are responsible for vision. Dopamine participates in the restructuring of the retina from a state of dark to a state of light adaptation. Sympathetic nerves innervating the retinal vasculature contain noradrenaline [139]. Recently, data have emerged on the participation of dopamine and noradrenaline in controlling angiogenesis in retinopathy of prematurity [140].

An interesting fact is that neuronal catecholamine synthesis is acutely enhanced by ascorbic acid [64,65].

In human prenatal development, catecholamines (dopamine, noradrenaline, and adrenaline) were shown in the vitreous on 17 and 18 weeks of gestation [128]. The presence of catecholamines has also been shown in the vitreous [141] and anterior chamber [142] fluids of adult humans.

Thus, catecholamines, being neurotransmitters, play an important physiological role in establishing interneuronal connections and, at the same time, provide antioxidant protection of neurons during their development.

### 3.6. Erythropoietin

**Figure 6:**
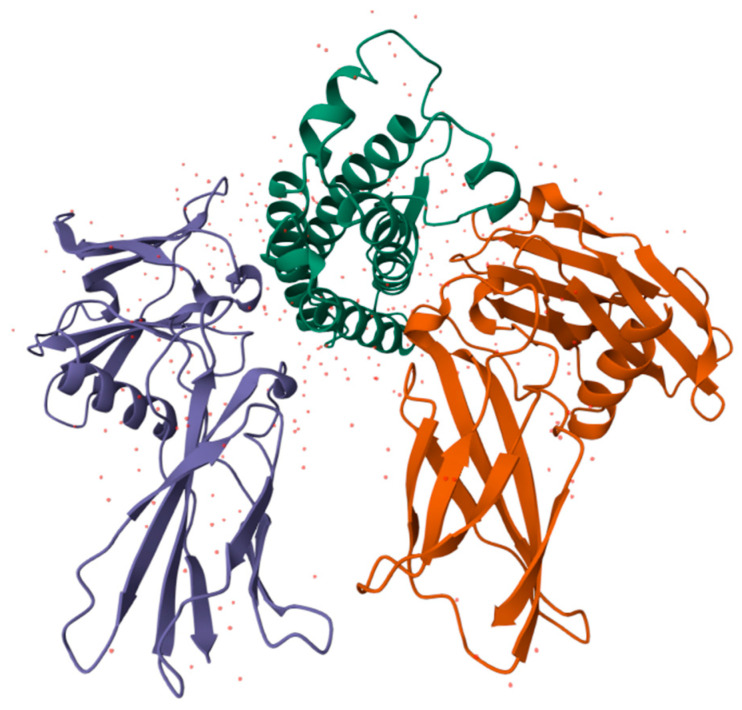
Structure of human EPO molecule (from [146]), an active antioxidant. It contains three N-linked and one O-linked side chains of acidic oligosaccharides.

Erythropoiesis, the process by which red blood cells are produced in response to hypoxia, is mostly regulated by the hormone erythropoietin (EPO). In order to increase the mass of red blood cells and preserve vascular oxygen homeostasis, it promotes the survival, growth, and differentiation of erythrocytic progenitors. Additionally, it has nonhematopoietic qualities such as antioxidant, angiogenic, neuroprotective, anti-apoptotic, and stem cell-modulatory activities [143,144]. With three N-linked and one O-linked side chains of acidic oligosaccharides, EPO is a 30.4 kDa glycoprotein composed of 165 amino acids (Figure 6) [145].

It is formed from a polypeptide that is encoded by the EPO gene, and then cleaved and glycosylated [147,148]. EPO is an active antioxidant and efficiently reacts with HO^•^ and ROO^•^ radicals [149,150,151,152].

Agents induced by oxidative stress can be blocked by EPO. Hydrogen peroxide has been shown to cause an overexpression of EPO, which lowers glutamate and ROS [153]. EPO may also have a direct antioxidant effect by promoting iron depletion, which lowers iron-dependent oxidative damage, and regulating glutathione peroxidase and HO-1 [149].

Kidney cells are the primary producers of EPO, which is secreted into the bloodstream and reaches the bone marrow, where it promotes the differentiation of hematopoietic stem cells into red blood cells [154]. EPO and its receptors have been found in extra-hematopoietic tissues, such as retinal tissue and retinal pigment epithelium (RPE) [155,156,157,158,159,160].

Neural crest- and neuroepithelium-derived cells release EPO throughout embryonic life to encourage erythrocyte differentiation in the yolk sac [161,162].

Hepatocytes and interstitial cells surrounding the liver central vein serve as the main locations for EPO synthesis during fetal life [163,164,165,166].

The tissue of the central nervous system also produces EPO, and the majority of the cells of the central nervous system, such as neurons, astrocytes, and microglia, express the EPO receptor (EPO-R) homodimer [167,168]. Additionally, the retina and vitreous exhibit Epo and Epo-R expression [157,169].

By reducing inflammatory response, EPO was shown to have a protective impact in the brain [170], and the retina experiences a similar effect. In the retina, EPO demonstrates resistance to ischemia, degeneration, permeability, inflammation, and damage from oxidative stress. Additionally, EPO is thought to be an angiogenic, endothelium-protective, and neuroprotective factor [171].

Patel et al. showed the presence of EPO in the vitreous and serum, as well as EPO mRNA in the retina, throughout prenatal human development from 12–14 to 21–24 weeks of gestation [172]. With increasing gestational age, the EPO concentration increased in the vitreous (from a median of 7.5 mU/mL at 12–14 weeks to a median of 26.8 mU/mL at 21–24 weeks) and serum, being significantly greater in the vitreous than in serum. EPO mRNA also increased in the fetal retina with age. This study indicates their direct correlation and that EPO plays a major role in the development of the visual system.

Therefore, EPO plays a crucial role in eye development, particularly in the retina, where it acts as an antioxidant with neuroprotective and angiogenic effects. It is involved in regulating cell fate, protecting against damage from hypoxia, and promoting neuronal survival and differentiation [171,172,173,174].

### 3.7. Albumin

**Figure 7:**
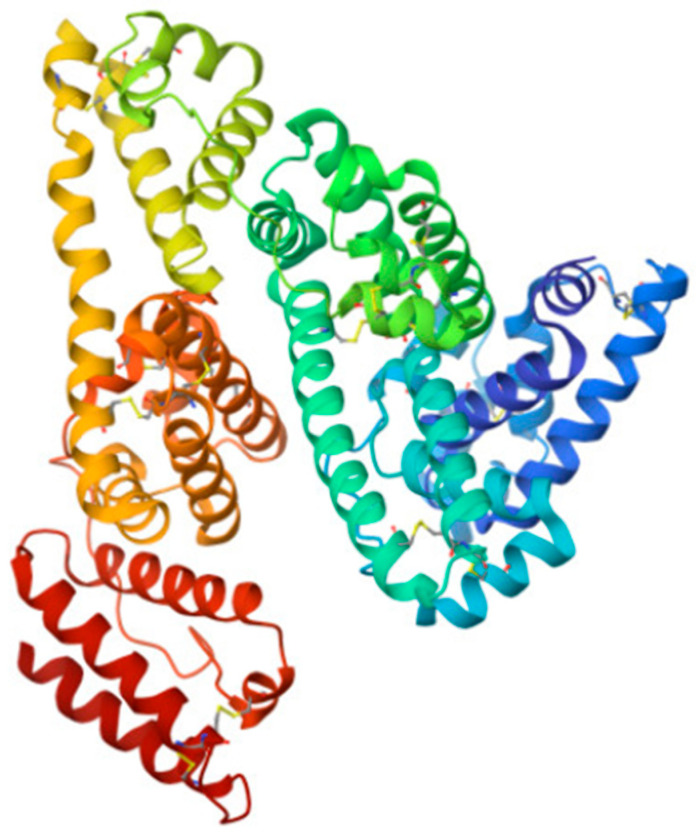
Structure of HSA—the main protein in the body (from [177]). It mainly performs transport functions and is also an antioxidant.

The main protein in the body throughout human ontogenesis is human serum albumin (HSA), a globular protein having a molecular mass of about 66 kDa, which is the main component of human blood serum [84,175,176] (Figure 7).

In the healthy vitreous, it makes up about 80% of the average protein concentration [178,179].

In the fetal vitreous, albumin was detected from the 10th to the 31st week of gestation. The maximum albumin concentration was reached at the 17th week and was equal to 211 µmol/L, after which the albumin concentration decreased, attaining a minimum value of 2.9 µmol/L at the 28th–31st weeks, which, on average, corresponds to the concentration in an adult. The total albumin content in the vitreous reaches its maximum value of 1.4 mg at the 20th–21st week of prenatal development and then decreases to 0.12 mg by the 31st week [180,181,182].

Albumin has important multifunctional properties: it creates the oncotic pressure of circulating blood plasma [84,183], it is the main carrier protein in the body [84,184,185,186,187], and it has antioxidant properties [183,188,189,190]. The antioxidant activity of HSA includes scavenging hydroxyl radicals by its reduced cysteine residue (Cys34), scavenging peroxynitrite by its thiol (-SH) group, and binding potentially ROS-generating ligands (especially the transition metal ions), thereby reducing their activity. Methionine residues (six in HSA) could also serve as a ROS scavenging system [188]. An important role of HSA also lies in the transport of a number of antioxidants (for example, carotenoids and bilirubin) throughout the body.

The oncotic (colloid osmotic) pressure created by albumin is essential to prevent the loss of liquid from the blood vessels, which helps keep an appropriate volume and pressure of blood. Thus, HSA can control the distribution of water, different ions, and molecules in various tissues of the body [191,192,193]. Serum albumin is a source of oncotic pressure in the growing vitreous during fetal life. This pressure helps to maintain the normal formation of the developing eye by contributing to the total (osmotic) intraocular pressure.

The unique property of albumin is its transport function: it binds and transports over the body many types of important biomolecules: polyunsaturated fatty acids, antioxidants, hormones, vitamins, cytokines, etc. [84].

Thus, albumin in the vitreous is essential for normal eye development.

### 3.8. Alpha-Fetoprotein

**Figure 8:**
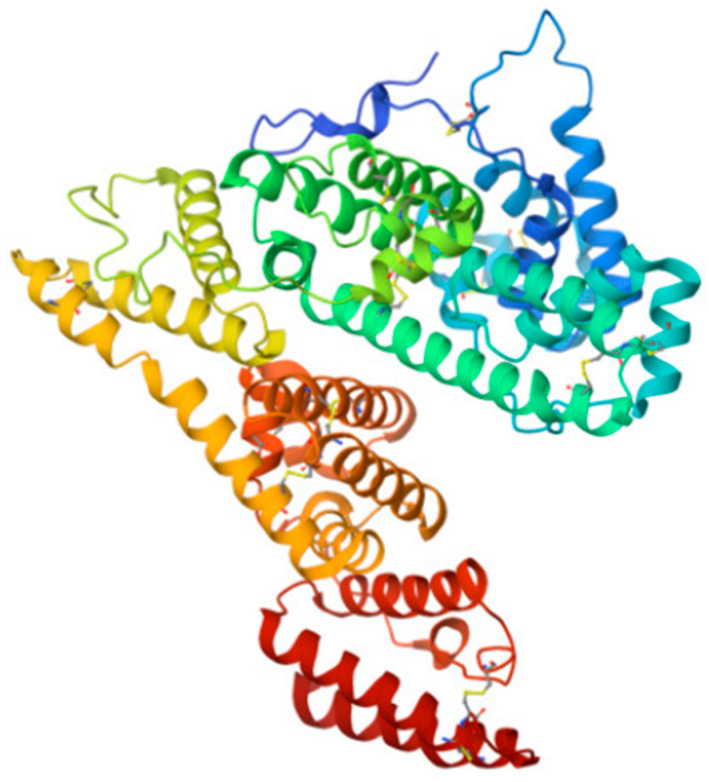
Structure of AFP molecule (from [197]). As HSA, it carries out transport and antioxidant functions.

Alpha-fetoprotein (AFP) is a specific blood serum protein of human embryos and fetuses. In adults, AFP is observed only in trace amounts. AFP is a member of the albumin family; its structure and functions are similar to those of serum albumin [84,175,176,194,195,196] (Figure 8).

It was shown in [182,198] that AFP was present in the human fetal vitreous between the 14th and 31st weeks of pregnancy. The maximum concentration of AFP in the vitreous occurs at 17th week of gestation (17.39 mg/mL, that is 252 µmol/L), then it begins to decrease (6.13 mg/mL, that is 89 µmol/L, at 22nd week) and after 26 weeks it decreases sharply, reaching a minimum (trace) value by 31 weeks. Thus, significant values of AFP concentration occur in the first and second trimesters of development.

AFP has a number of special properties that are important for early prenatal development: (1) immunosuppressive properties, which protects the fetus from the maternal immune response [199,200]; (2) a number of properties common with polypeptide growth factors [201]; (3) it is involved in sexual differentiation of the brain (shown in rodents) [202,203].

Like albumin, AFP creates oncotic pressure in the fetal bloodstream [84] and is responsible for the intraocular pressure of the fetus.

An important role of AFP is the transport and delivery to developing tissues of polyunsaturated fatty acids [194,204,205], lutein [182,206], bilirubin [182], and other molecules.

AFP also has the properties of an effective antioxidant, exhibiting synergistic antioxidant activity in the presence of estradiol [190].

In the developing vitreous, the retina (particularly the developing macula) and the lens, as well as the vitreous itself, are successfully protected from oxidative stress by albumin and AFP with bound lutein, which have antioxidant properties. Bilirubin in physiological concentrations, bound to albumin and AFP, forms an important antioxidant complex that protects developing eye tissues from oxidative stress [182].

## 4. Discussion

**Figure 9:**
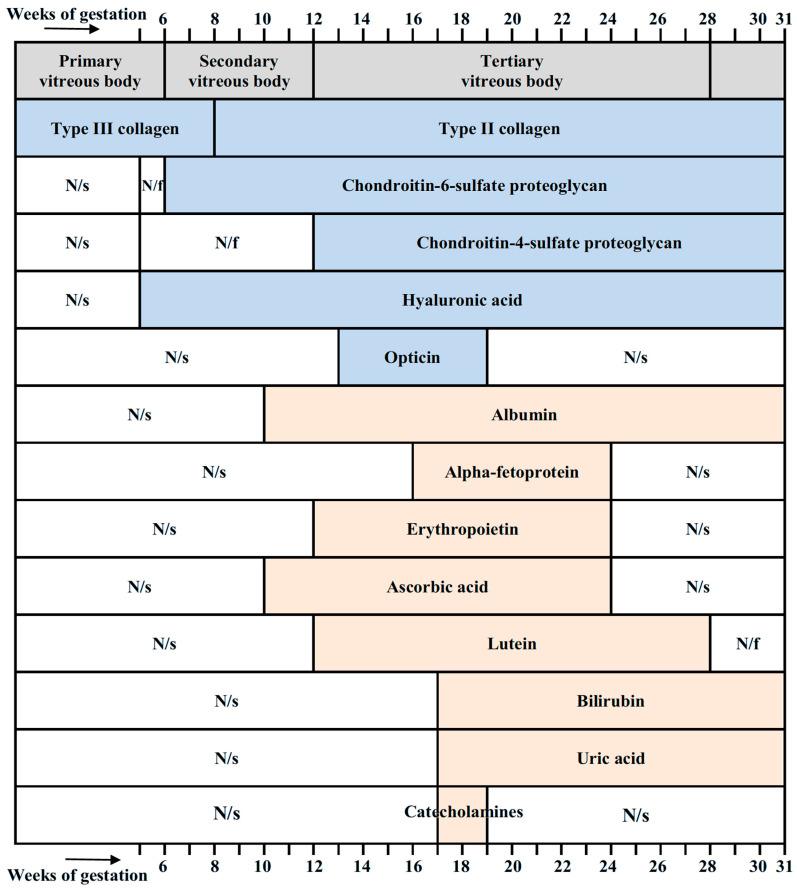
Stages of development of the vitreous (according to Ida Mann [11]) and its molecular components with their timing. Collagens [21], chondroitin-sulfate-proteoglycans [21], hyaluronic acid [21,23], and opticin [24] are structural components of the vitreous and are not antioxidants. Albumin [180,181,182], alpha-fetoprotein [182,198], erythropoietin [172], ascorbic acid [58], lutein [76], bilirubin [117], uric acid [128], and catecholamines [128] have antioxidant properties. N/s, not studied; N/f, not found.

Figure 9 summarizes essential molecular components of the vitreous in the developing human eye, together with their timing. Collagens, chondroitin-sulfate-proteoglycans, hyaluronic acid, and opticin are components necessary for maintaining the structural integrity of the vitreous. Other molecular components shown in Figure 9 have antioxidant properties.

The diversity of antioxidants found in the developing vitreous may be explained by the enhancement of their effectiveness upon mutual interaction—synergism, which is observed in many cases. In particular, ascorbic acid protects the power of other antioxidants such as α-tocopherol by reducing their oxidized radical forms [37,57] and can increase the antioxidative ability of uric acid in blood plasma by reparation of urate radicals formed upon oxidative stress [125]. Ascorbic acid also increases the antioxidative effect of neuronal catecholamines by sharp enhancement of their synthesis [64,65]. Alpha-fetoprotein demonstrates synergism in its antioxidant ability with estradiol [190].

Albumin and AFP, with their transport function, help the action of a number of antioxidants (for example, carotenoids, bilirubin) by delivering them to the protected tissue. Bilirubin in physiological concentrations, bound to albumin and AFP, forms an important antioxidant complex that protects developing eye tissues from oxidative stress [84,101].

**Figure 10:**
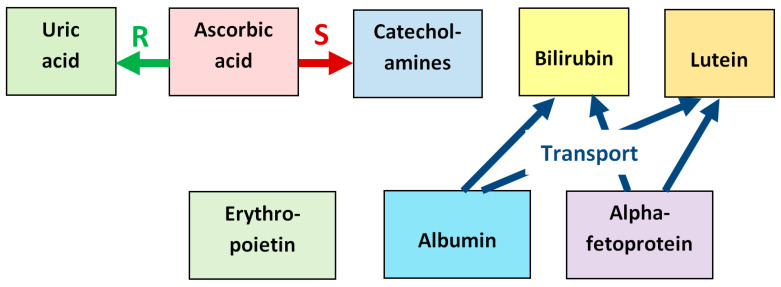
Synergistic relationship between the antioxidants found in the fetal vitreous. Ascorbic acid reduces the oxidized urate radical formed in reactions with ROS, which repairs the initial uric acid molecule (label “R”). Ascorbic acid also stimulates the synthesis of neuronal catecholamines, which increases their antioxidative effect (label “S”). Albumin and alpha-fetoprotein have transport functions with respect to several antioxidants, in particular, bilirubin and lutein, delivering them to target tissues (label “Transport”).

The synergistic relationship between the antioxidants found in the fetal vitreous is illustrated in Figure 10, which schematizes reparation of oxidized uric acid by ascorbic acid, stimulation of synthesis of neuronal catecholamines by ascorbic acid, and transport functions of albumin and alpha-fetoprotein with respect to bilirubin and lutein.

The data on antioxidants found to date in the vitreous of the developing eye are summarized in Table 1.

The total content of antioxidants in the vitreous can be roughly estimated by the photochemical method—quenching of the triplet state of riboflavin [207], since almost all antioxidant molecules are electron donors that quench triplet riboflavin molecules. This estimation showed that the overall content of electron donor molecules in the vitreous of human fetuses of 24 and 31 weeks of gestation was about 990 and 940 µmol/L, respectively [128]. These concentrations are lower than those estimated by the same method in the vitreous of adult humans (~1400–~3300 µmol/L [128]). Note that these values are probably lower estimates of antioxidant concentrations in the vitreous, since not all antioxidants effectively quench the riboflavin triplet state (for example, carotenoids do not quench [208]).

Summarizing the data on the antioxidant concentrations given in Table 1 and comparing them with the estimates made above (for 24–31 weeks of gestation), it can be understood that the actual total content of antioxidants in the vitreous is probably much higher than the data given in the table. This means that a number of antioxidants in the fetal vitreous have not yet been detected and require further research. In addition, the antioxidant status of the primary vitreous (the first 6 weeks of gestation) has not been studied. Antioxidant molecules in the secondary vitreous (6–12 weeks) have also been insufficiently studied. Since the initial formation of the main structures of the eye occurs at these stages, studying the antioxidant status at these times is especially important for understanding the role of antioxidants in normal embryonic development of the eye. It is also important to expand the study of the molecular spectrum of antioxidants.

The functions of the presented molecules are much wider than just antioxidants (some other functions are illustrated in Figure 10), and their role can be timed to the events occurring in the course of eye development. This is possibly associated with the transient presence of some molecules (lutein, AFP), or a decrease in their concentration with the age of the fetus (albumin, bilirubin), or a tendency to increase concentration (ascorbic acid, erythropoietin). Note that the concentration of a number of antioxidant molecules in the vitreous falls by the 28th week of pregnancy. This indicates a change in the antioxidant spectrum at this time and may be a key moment in the prenatal development of the human eye.

It is known that an imbalance between the production of ROS and weak antioxidant defense is one of the main factors in the pathogenesis of many eye diseases, some of which may develop during pregnancy [209,210]. To ensure therapy for such diseases, it is important to maintain the necessary redox balance with the help of antioxidant administration. Of the molecules found in the fetal vitreous, lutein can be useful in the treatment of oxygen-induced retinopathy [211], and EPO against various eye diseases [212]. Bilirubin nanoformulations are also promising in treating ROS-induced illnesses [213]. This suggests possible administration of these antioxidants (and ascorbic acid) for the prophylactic of ocular diseases during pregnancy. In this regard, finding new antioxidant molecules in the fetal vitreous could create an additional opportunity. Of course, such applications need further investigations and clinical trials.

## 5. Conclusions

This review presents molecules found in the vitreous of the eye throughout pregnancy, which have antioxidant activity. Until now, the antioxidant role of these molecules in the vitreous of the human fetal eye has not been considered. Compared to the adult human vitreous, in which 17 types of antioxidant molecules were identified [5], on the basis of the literature sources, we have not been able to reveal as many types of antioxidants in the vitreous of human fetuses. However, this does not mean that there is really a small variety of them in the vitreous of the developing eye.

The fact that there are probably many more antioxidants than have been found so far is indicated, in particular, by the photochemical estimation of the overall concentration of electron donors in the vitreous of human fetuses by quenching the triplet state of riboflavin [128]. Thus, further research is needed to find new antioxidant molecules in the vitreous of the developing eye and to study their role in eye development. It is especially important to study the antioxidant status of the vitreous at the earliest stages of its development. Antioxidants found in the fetal vitreous suggest their possible administration for the prophylactic of ocular diseases during pregnancy. Finding new antioxidant molecules could create an additional opportunity in this regard.

## Figures and Tables

**Table 1:** Antioxidant molecules found in the vitreous of the human fetal eye.

Molecule	Gestation Age Studied, Weeks	Concentration, µmol/L (Age)	Main Functions	Significance in Eye Development	Refs.
Ascorbic acid	10–24	18 → 114 (increases with age)	ROS scavenging, preventing lipid peroxidation, participation in collagen synthesis, and antiangiogenic	Structural integrity, tissue protection, involvement in cellular proliferation and differentiation	[58]
Lutein	12–31	1.6–1.8 (maximum, at 17–22 weeks); 0.007 (24–28 weeks); no (30–31 weeks)	Singlet oxygen quenching, neuroprotection, regulation of apoptosis, and maintaining tissue homeostasis	Retina/macula development, oxidation defense	[76,95,96,97,98]
Bilirubin	17–31	Average 8.67 (17–19 weeks); average 1.37 (20–31 weeks)	ROS scavenging, Nrf2 activation, anti-inflammatory, immunomodulatory, cytoprotective, and neuroprotective	Supports neuronal differentiation, membrane protection	[59,106,107,108,109,110,111,117]
Uric acid	17–31	37–248	ROS scavenging, neuroprotection, stimulation of immune system by activating T cells	Redox balance, tissue protection, inhibits oxidation of hyaluronic acid by ascorbate	[123,124,125,126,127,128]
Catechol-amines	17, 18	Present (not measured quantitatively)	Antioxidant, vascular, and neuronal regulation	Neural and vascular development	[128]
Erythro-poietin	12–24	Average 7.5 mU/mL (12–14 weeks); average 26.8 mU/mL (21–24 weeks)	Erythropoiesis, antioxidant, neuroprotection, and angiogenesis	Oxygen regulation, tissue survival	[143,144,172]
Albumin	10–31	211 (maximum, 17 weeks); 2.9 (minimum, 28–31 weeks)	Transport, antioxidant, and maintaining oncotic pressure	Molecular transport, maintaining intraocular pressure	[84,176,180,181,182]
Alpha-fetoprotein	14–31	252 (maximum, 17 weeks); 89 (22 weeks); trace (by 31 weeks)	Immunosuppression, fatty acid transport, antioxidant, and maintaining oncotic pressure	Tissue protection, growth regulation, maintaining intraocular pressure, molecular transport, function of cytokine in development	[175,182,194,195,196,198,201]

## Data Availability

Not applicable.
